# Navigating a new era in cardiovascular disease epidemiology: big data, artificial intelligence and the imperative of disability inclusion

**DOI:** 10.3389/fepid.2026.1871078

**Published:** 2026-07-09

**Authors:** Theophilus I. Emeto

**Affiliations:** Public Health and Tropical Medicine, College of Medicine and Dentistry, James Cook University, Townsville, QLD, Australia

**Keywords:** algorithmic fairness, artificial intelligence, big data, cardiovascular epidemiology, disability, FAIR data principles, health equity, polygenic risk score

## Abstract

Cardiovascular disease (CVD) remains the leading cause of premature global mortality and one of the largest contributors to disability-adjusted life years lost. Over the past decade, the field has been transformed by the convergence of population biobanks, deep learning applied to imaging and electrocardiography, polygenic risk scores, wearable biosensors, and methodological advances in causal inference and target trial emulation. These innovations are reshaping precision public health for the general population. Yet the gains have not been equitably distributed. People with disability (PwD), comprising approximately 16 per cent of the global population and recognised by the United States National Institute on Minority Health and Health Disparities as a population experiencing health disparities are systematically under-represented in clinical trials, biobanks, electronic health records and the artificial-intelligence (AI) models trained upon them. Their cardiovascular health is therefore both worse and less precisely characterised than that of the general population. This article maps the key methodological vectors of change in CVD epidemiology, explains why each has so far failed to reach PwD, and presents a layered, defendable framework for disability-inclusive big-data and AI-enabled CVD research. It argues that disability inclusion is not a peripheral equity concern but a stress-test for the validity, generalisability and ethical legitimacy of the entire precision-cardiovascular enterprise.

## 1 Introduction

Cardiovascular disease (CVD) remains the foremost cause of premature mortality worldwide and one of the largest contributors to global disability-adjusted life-years (DALYs) lost; in 2021, ischaemic heart disease and stroke ranked among the top five sources of DALYs in 190 of 204 countries (1). Notwithstanding this enduring burden, CVD epidemiology is in the midst of its most significant methodological reconfiguration since the Framingham era. Three intersecting currents are reshaping the discipline: the integration of multi-omic data with deep clinical phenotyping, including genome-wide polygenic risk scores (2, 3); the application of machine and deep learning to high-dimensional cardiovascular signals such as electrocardiography and cardiac imaging (4); and the maturation of population-scale linked-data infrastructures, including the United Kingdom Biobank (5), the United States *All of Us* Research Program (6) and Australian linked health-administrative collections, which together have moved the field from static, linear risk-factor analysis towards dynamic, individualised risk modelling under the banner of precision public health.

These advances have not been distributed equitably. An estimated 1.3 billion people, or 16 per cent of the global population, live with a significant disability and experience earlier death, poorer health and greater limitations in everyday functioning than their non-disabled peers, despite holding the same right to the highest attainable standard of health (7). In September 2023, the United States National Institute on Minority Health and Health Disparities (NIMHD) formally designated people with disability (PwD) as a population experiencing health disparities for the purposes of National Institutes of Health-funded research (8), and leading clinical journals have concurrently reframed structural ableism as a determinant of health (9). Taken together, these developments mark an inflection point that the present article takes as its starting position.

The CVD implications of this evidence gap are substantial. PwD bear a disproportionate CVD burden, with prevalence of heart disease and stroke approximately three to four times that of people without disability (10, 11), and with standardised mortality ratios for circulatory disease in adults with intellectual disability of approximately 2–5 in recent population-based and record-linkage analyses (12, 13). Yet they remain markedly under-represented in the very datasets and clinical trials that drive contemporary discovery: a structured analysis of trial eligibility criteria found that disability-related exclusions are widespread and frequently lack scientific or ethical justification, threatening the generalisability of the precision-CVD evidence base (14).

This article uses the cardiovascular health of PwD as a stress test of the contemporary epidemiological toolkit. Four objectives structure the paper: (i) to summarise the methodological innovations currently reshaping CVD epidemiology; (ii) to interrogate why these innovations have largely bypassed PwD; (iii) to propose a layered architecture for disability-inclusive, big-data- and artificial-intelligence (AI) enabled cardiovascular research; and (iv) to articulate a tractable research agenda for the next five years.

Throughout, the term “people with disability (PwD)” is used in alignment with the World Health Organization's *International Classification of Functioning, Disability and Health* (ICF), which conceptualises disability as the product of an interaction between a person's functional capacity and their physical, social and attitudinal environment (15). Where data sources cited in this article apply narrower or older operational definitions, this is made explicit.

### 1.1 Methods and search strategy

This article is a narrative review with a conceptual-framework component, prepared in response to an invitation from the Research Topic editors and structured to inform methodological and policy reform rather than to perform an exhaustive systematic synthesis. The article does not apply an exhaustive, pre-registered protocol with formal risk-of-bias appraisal, nor does it apply any form of quantitative pooling. The article purposively selects and interprets the evidence base to advance a conceptual argument, thus the structured search described below is reported to maximise transparency and reproducibility, not to support any claim of systematic completeness. Nonetheless, a targeted, reproducible literature search was conducted between 01 March 2024 and 30 April 2026 across PubMed/MEDLINE (primary database, ovid) and Scopus (citation tracking). This was supplemented by direct searches of the institutional websites of the World Health Organization (WHO), the United States National Institutes of Health and National Institute on Minority Health and Health Disparities, the Australian Institute of Health and Welfare (AIHW), the Australian Bureau of Statistics (ABS), and the Lancet Countdown on Health and Climate Change, for authoritative grey-literature sources. The search combined three concept blocks linked by Boolean AND, each using both Medical Subject Headings (MeSH) and free-text title and abstract terms: (i) cardiovascular epidemiology and methods (cardiovascular diseases OR coronary artery disease OR stroke; combined with cohort studies, biobank, electronic health records (EHR), machine learning, artificial intelligence, polygenic risk score, target trial emulation, wearable electronic devices); (ii) disability and health equity (disabled persons, intellectual disability, International Classification of Functioning, health equity, health disparities, structural ableism, algorithmic fairness); and (iii) context-specific terms for the COVID-19 (SARS-CoV-2, vaccination), environmental (air pollution, particulate matter, climate change) and data-governance (Findability, Accessibility, Interoperability, and Reuse of digital assets (FAIR) principles, federated learning) subsections.

Searches were restricted to peer-reviewed publications in English from 01 January 2014 to 30 April 2026, with the exception of foundational methodological and policy sources (the WHO International Classification of Functioning and the FAIR Guiding Principles), which were retained on conceptual grounds. Reference lists of all included articles were hand-searched and forward citation tracking was performed in Scopus and Google Scholar for landmark papers. Inclusion was prioritised, in descending order, for: (i) peer-reviewed primary studies in high-impact general medical, cardiovascular or epidemiological journals, particularly large population-based cohort studies, biobank analyses, target trial emulations and systematic reviews with meta-analysis; (ii) consensus statements and population-prevalence reports from the WHO, the Global Burden of Disease Study, the AIHW, the ABS and the Lancet Countdown; (iii) methodological papers establishing the conceptual frameworks invoked in the review (target trial emulation, polygenic risk scoring, fairness-aware machine learning, the Transparent Reporting of a multivariable prediction model for Individual Prognosis Or Diagnosis (TRIPOD)+AI reporting standard and the FAIR data principles); and (iv) Australian and Asia-Pacific evidence where directly relevant to the local policy framing. Records were excluded if they were conference abstracts without full peer-reviewed publication, opinion pieces lacking primary data or framework contribution, or preprints not subsequently peer-reviewed by April 2026. Every citation was independently verified against PubMed, the publishing journal's homepage and the Digital Object Identifier (DOI) system; references that could not be authenticated, or whose content did not support the claim attributed to them, were removed and replaced. To document the selection process transparently, the structured search identified 1,604 records from PubMed/MEDLINE and Scopus after de-duplication, with a further 238 records from grey-literature and citation tracking, giving 1,842 records screened at title and abstract; 142 full-text records were assessed for eligibility; and 39 sources were ultimately retained for citation in the synthesis (Supplementary Figure S1). Consistent with the narrative design, no formal risk-of-bias appraisal or quantitative meta-analysis was undertaken; the flow diagram is provided as a transparency aid and does not constitute a claim of PRISMA-compliant systematic-review conduct.

## 2 Methodological innovations and the digital frontier

This section synthesises four current interlocking technologies that are reshaping CVD epidemiology and constitute the analytic toolkit drawn upon throughout the remainder of the article.

### 2.1 Predictive analytics on linked data

In contemporary CVD epidemiology, “Big data” refers to data infrastructures and analytic operations characterised by the five Vs: (i) Volume, multi-million-record linkages across national administrative datasets (UK Biobank *n* > 500,000; the United States *All of Us* Research Program *n* > 800,000; the Australian Multi-Agency Data Integration Project); (ii) Velocity, continuous biosensor and EHR streams that enable near-real-time phenotyping; (iii) Variety, integration of structured clinical codes, unstructured clinical text via natural language processing, imaging signals via convolutional neural networks, genomic data, wearable signals and patient-reported outcomes; (iv) Veracity, necessitating provenance metadata, data-quality scoring and the FAIR principles; and (v) Value, the capacity to detect rare CVD phenotypes, validate causal-inference estimates with sufficient statistical power, and generate person-level rather than population-average predictions. The big-data processing pipeline (data ingestion, harmonisation, feature engineering, modelling and translation) is made operationally explicit in the architecture proposed in Section 4.4 and depicted in Figure 2.

Population-scale linkage of electronic health records (EHR), administrative claims, and biospecimen data has enabled discovery at a granularity that was inaccessible a decade ago. UK Biobank analyses of more than 220,000 individuals have, for example, permitted long-horizon prospective evaluation of post-COVID cardiovascular risk and ABO-blood-group interactions (16). Wearable biosensor data are increasingly integrated with clinical records to produce continuous-time phenotypes of arrhythmia, sleep and physical activity, complementing episodic clinical observation with high-frequency physiological signal (4).

### 2.2 Polygenic risk and the heritability question

The transition from candidate-gene studies to genome-wide polygenic risk scores (PRSs) has refined the genetic stratification of coronary heart disease, atrial fibrillation and other complex CVD phenotypes (2, 3), and PRSs are now being incorporated into clinical-trial protocols for primary prevention. The “missing heritability” problem which is the discrepancy between trait heritability inferred from family studies and the variance explained by common variants reaching genome-wide significance, is increasingly understood to reflect a combination of rare variants of larger effect, structural variation, gene–gene and gene–environment interactions, and ancestry-specific architecture (3). The clinical utility of PRSs in non-European-ancestry populations remains a recognised limitation (2, 3), and disability-stratified PRS performance has not been routinely reported.

### 2.3 Causal inference and target trial emulation

In parallel, target trial emulation has matured into a defendable framework for drawing causal inferences from observational data when randomised trials are infeasible (17). The approach specifies the protocol of a hypothetical randomised trial, eligibility, treatment strategies, assignment, follow-up, outcome and analysis plan, and emulates each component using observational data, with explicit handling of immortal-time bias, time-zero alignment and post-baseline confounding. The methodology is particularly germane to disability research, in which pragmatic randomised trials are scarce, ethically constrained and frequently underpowered.

### 2.4 Artificial intelligence in cardiovascular care

AI-enabled tools have demonstrated diagnostic performance comparable to, and in selected signal-processing domains exceeding, that of expert clinicians: convolutional neural networks applied to twelve-lead electrocardiograms can detect asymptomatic left-ventricular dysfunction; deep-learning models applied to cardiac magnetic resonance imaging quantify ventricular function and fibrosis at scale; and large language models are being trialled for clinical documentation and decision support (4, 18). A recent systematic review of AI in cardiovascular diagnostics reinforces this picture: across 14 high-quality studies appraised with the Quality Assessment of Diagnostic Accuracy Studies 2 (QUADAS-2) and Prediction model Risk Of Bias ASsessment (PROBAST) tools, AI-based diagnostic tools achieved reported area-under-the-curve values ranging from 0.804 to 0.991 across electrocardiography, cardiac imaging and predictive risk modelling, while the same review emphasised the scarcity of external validation, a limitation that maps directly onto the disability-stratified reporting argued for in this article (19). Davenport and Kalakota's contemporaneously cited overview of AI's current and prospective roles across health care (20), flags persistent issues of generalisability, regulatory approval and bias. These are issues to which this article returns to in Section 4.

Five principal AI architecture families currently dominate CVD applications and warrant brief technical description, given their differing analytic affordances and risk profiles. *Convolutional neural networks* (CNNs) extract spatial features from images and 1-D physiological signals, and underlie state-of-the-art electrocardiogram, echocardiography and cardiac magnetic resonance interpretation. *Recurrent neural networks and transformer-based sequence models* handle longitudinal EHR data and time-series biosensor signals, capturing temporal dependencies that conventional regression cannot. *Gradient-boosted decision-tree ensembles* (e.g., XGBoost, LightGBM) offer state-of-the-art tabular risk prediction with comparatively transparent feature importance. *Graph neural networks* encode multi-relational EHR phenotyping, linking diagnoses, medications and procedures. *Large language models* are being trialled for clinical-note phenotyping and decision-support summarisation. Each architecture trades the gains of non-linear interaction modelling, automatic feature extraction and multi-modal fusion against losses of interpretability, generalisability and calibration stability, and against the disability-related fairness deficits central to this review.

These tools are also increasingly framed within a population and public-health perspective: a 2025 review of AI applications in cardiovascular health following the COVID-19 pandemic argues that machine- and deep-learning methods can strengthen post-pandemic cardiovascular surveillance, risk prediction and service delivery, while cautioning that data quality, algorithmic bias and equitable access remain decisive constraints (21). Conceptually distinct from these signal-level applications, cardiovascular “digital twins” are an emerging research paradigm with proof-of-concept demonstrations in personalised electrophysiology, valve modelling and *in-silico* clinical trials (18). Digital twins are not yet validated for routine clinical decision making, and the term is used here descriptively rather than prescriptively.

## 3 The COVID-19 catalyst and the new risk landscape

The SARS-CoV-2 pandemic functioned as both an accelerant of methodological adoption and a clarifying test of the field. Three findings deserve particular emphasis, and a fourth, the disability dimension that pandemic research has largely missed, frames the agenda of subsequent sections.

### 3.1 SARS-CoV-2 directly infects the coronary vasculature

Eberhardt and colleagues, using post-mortem coronary tissue from severe COVID-19 cases together with *ex-vivo* infection of human atherosclerotic vascular explants, demonstrated that SARS-CoV-2 viral RNA is detectable in, and replicates within, coronary atherosclerotic lesions, preferentially targeting plaque macrophages and foam cells via a neuropilin-1-dependent pathway, and inducing a pro-atherogenic cytokine response (22). This work establishes a plausible mechanistic basis for the elevated risk of acute coronary syndrome and stroke observed for up to one year after infection.

### 3.2 COVID-19 as a coronary artery disease risk equivalent with caveats

Hilser and colleagues used UK Biobank data, comparing 10,005 incident COVID-19 cases (8,062 polymerase-chain-reaction-confirmed and 1,943 ICD-10-coded) with 217,730 population controls and 38,860 propensity-score-matched controls (16). Hospitalised COVID-19 was associated with a more than threefold higher risk of major adverse cardiovascular events (MACE) over follow-up exceeding 1,000 days, with a magnitude approximating that of established coronary artery disease (CAD) risk equivalents such as type 2 diabetes mellitus and peripheral artery disease (16). The “CAD risk equivalent” framing applies most robustly to hospitalised cases and to a population skewed towards older adults of European ancestry; published correspondence has rightly counselled against extrapolation to milder infections in younger or more demographically diverse populations.

### 3.3 Vaccination and major cardiovascular events

Meister and colleagues conducted a target trial emulation using Estonian electronic medical record data from April 2021 to March 2023, evaluating pre-infection COVID-19 vaccination among adults aged 40–85 years (23). Among 33,554 individuals with subsequent SARS-CoV-2 infection (18,223 vaccinated; 15,331 unvaccinated), pre-infection vaccination was associated with a weighted incidence-rate ratio for MACE of 0.71 (95 per cent confidence interval 0.58–0.84) over twelve months. This finding is broadly concordant with a systematic review and meta-analysis of seasonal influenza vaccination by Cheng and colleagues, which pooled 63 studies of cardiovascular outcomes and reported a 26 per cent reduction in cardiovascular events among vaccinated relative to unvaccinated adults (pooled adjusted relative risk 0.74; 95 per cent confidence interval 0.70–0.78) (24). Both literatures remain susceptible to residual confounding by indication and the healthy-vaccinee bias, although target trial emulation explicitly addresses some but not all of these concerns.

### 3.4 What was missed: the disability dimension

PwD experienced disproportionate COVID-19 morbidity and mortality during the pandemic. Kuper and Smythe's meta-analysis of 56 prospective studies reported a pooled adjusted hazard ratio for COVID-19-related mortality of 2.7 (95 per cent confidence interval 2.4–3.2) compared with people without disability, rising to 3.3 in population-based samples and highest of all among adults with intellectual disability (25). Despite this, people with disability remain markedly under-represented in the post-acute sequelae cohorts and target-trial-emulation studies that now inform cardiovascular guidelines (25). This omission is not incidental: the linked-data infrastructures and emulation designs that delivered the rapid pandemic-era evidence base could, with relatively modest adaptation, mandatory ICF-coded disability indicators, accessible recruitment and consent procedures, and reporting of model performance stratified by disability status become the most powerful methodological assets available for closing the disability cardiovascular evidence gap.

## 4 The disability evidence gap and a framework for closure

### 4.1 The cardiovascular disparity and disability heterogeneity

PwD, approximately 1.3 billion individuals globally (7) and an estimated 5.5 million Australians (21.4 per cent of the population) according to the Australian Bureau of Statistics 2022 Survey of Disability, Ageing and Carers, up from 4.4 million (17.7 per cent) in 2018 (26), face a heightened CVD burden generated by a confluence of biological, behavioural, environmental and structural factors. Carroll and colleagues, analysing United States National Health Interview Survey data from 2009 to 2012, found that nearly half of working-age adults with disability were physically inactive and reported chronic disease prevalence three to four times higher than peers without disability (27). Krahn and colleagues subsequently synthesised the evidence to argue, successfully, that PwD meet the formal criteria for a health-disparity population (10).

Disability is, however, profoundly heterogeneous; CVD risk, the mechanisms of data-capture failure, consent practicalities, wearable usability and diagnostic-overshadowing risk all differ materially across disability domains, and no single analytic or policy solution will apply equally to all groups. People with intellectual disability bear the highest excess CVD mortality (standardised mortality ratios 2–5) (13, 25), with risks graded by severity, in a linked-registry analysis, hazard ratios for incident CVD were 1.14 (95% CI 1.01–1.30) for borderline or mild, 1.25 (1.01–1.54) for moderate, and 1.91 (1.47–2.48) for severe or profound intellectual disability (29). They also experience multimorbidity at rates nearly four times those of the general population, with implications for polypharmacy and antipsychotic-medication-related cardiometabolic risk (28). People with physical disability face disproportionate sedentary-behaviour-mediated CVD risk and require adapted physical-activity exposure measures; people with sensory disability face heightened diagnostic overshadowing in primary care, particularly where clinical communication is not adapted; people with psychosocial disability face CVD risk attributable to both antipsychotic-related metabolic effects and the structural consequences of social marginalisation; and people with neurodevelopmental disability may carry syndromic genetic architectures (e.g., trisomy 21, 22q11.2 deletion syndrome) in which standard polygenic risk score calibration cannot be assumed. People with multiple or co-occurring disabilities experience compounded barriers across all five domains. The proposed framework (Section 4.4) therefore emphasises disability-domain stratification rather than a single binary indicator.

Three contemporary population-based cohort analyses sharpen this picture. Wang and colleagues (29), using Danish national registries on 2,288,393 live-born singletons born between 1978 and 2016, reported a 24 per cent higher hazard of incident CVD in people with intellectual disability, with elevated risk evident in childhood and persisting into early adulthood (26). Cho and colleagues, using linked Korean National Disability Registration and National Health Insurance Service data, demonstrated higher risks of myocardial infarction and ischaemic stroke in adults with intellectual disability after adjustment for traditional cardiovascular risk factors (12). In Scotland, Rydzewska and colleagues, in a record-linkage cohort of 514,878 adults, reported materially higher all-cause and avoidable mortality among adults with intellectual disability than among matched general-population peers (13).

### 4.2 Why the methodological revolution has bypassed people with disability

Despite this evidence, PwD remain marginal in CVD discovery. Five mutually reinforcing mechanisms operate.

- **Trial exclusion.** Many CVD randomised trials apply cognitive, communication or capacity criteria that disproportionately exclude people with disability, often without explicit empirical or ethical justification (14).
- **Data invisibility.** Disability is rarely captured as a structured variable in electronic health records or population biobanks; where it is captured, definitions are inconsistent and rarely interoperable across jurisdictions.
- **Algorithmic bias.** Risk-prediction tools and clinical AI models are typically trained on cohorts that do not adequately represent disabled populations; published audits of medical machine-learning systems have repeatedly identified degraded performance, miscalibration and downstream allocation bias against under-represented groups (30, 31).
- **Diagnostic overshadowing.** New CVD symptoms in PwD are often misattributed to the underlying disability, leading to delayed diagnosis, under-treatment and consequent under-ascertainment in administrative datasets (9).
- **Wearable and digital exclusion.** Consumer wearables and remote-monitoring devices are validated principally on younger, non-disabled, ambulant populations; signal quality, motion-artefact handling and user-interface accessibility have rarely been formally evaluated in PwD.

**Figure 1:**
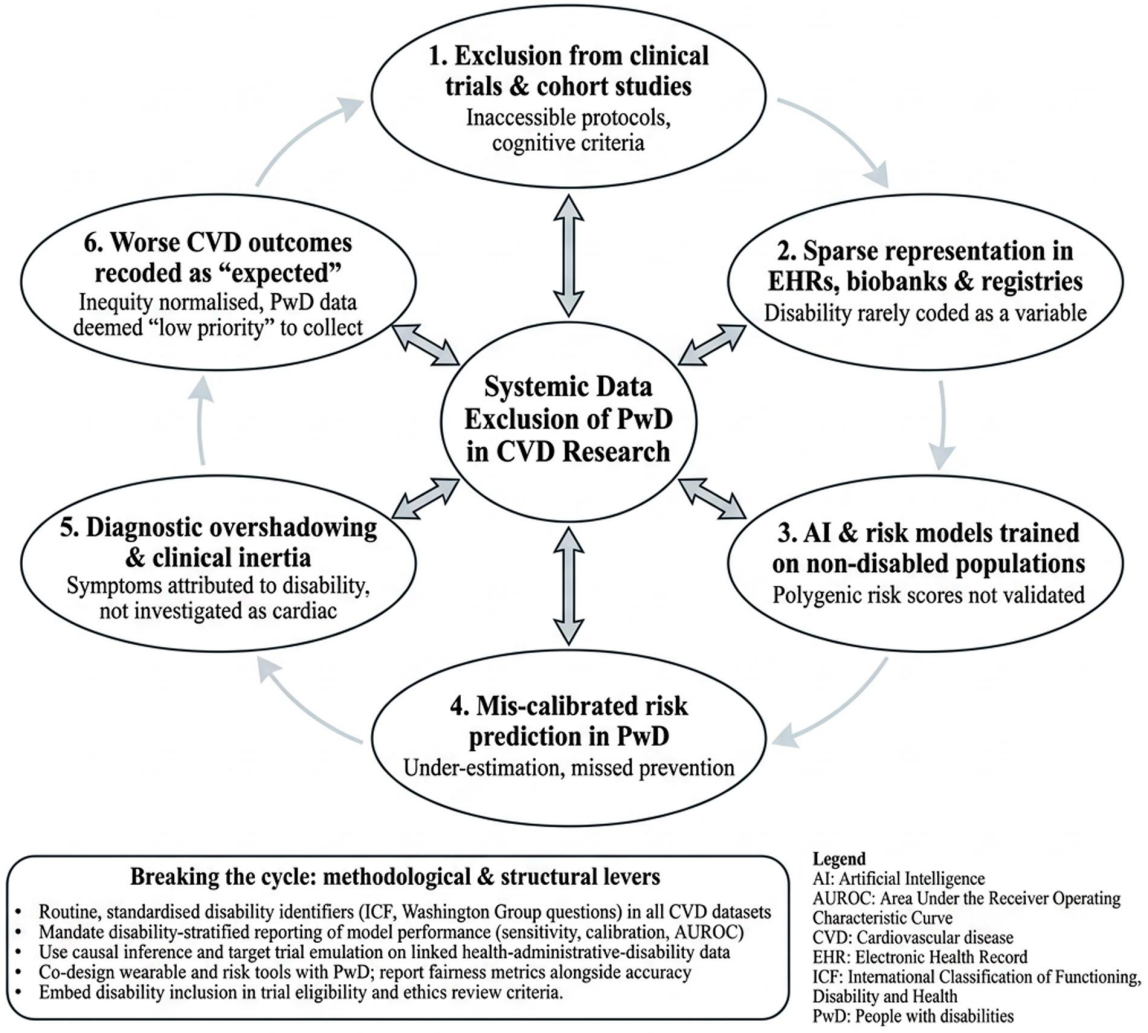
The self-reinforcing cycle through which exclusion from data perpetuates cardiovascular health inequities for people with disability, and the structural and methodological levers required to break it.

Together, these mechanisms constitute a self-reinforcing cycle (Figure 1): exclusion from trials produces sparse data, which yield biased models, which generate mis-calibrated risk estimates, which contribute to clinical inertia and worse outcomes, which are in turn re-coded as expected and used to justify continued non-investment. Breaking this cycle is the central methodological task of disability-inclusive CVD epidemiology.

### 4.3 Mapping innovations to gaps: a comparative summary

Table 1 Maps the five principal methodological innovations of contemporary CVD epidemiology to the disability-related research gap they currently exhibit, the consequence for validity and equity, and a proposed remedy. The table is intended to function as a reference summary for readers and as a structured agenda for funders and journal editors..

**Table 1:** Mapping methodological innovations to disability-related gaps and proposed remedies.

Innovation	Current use in mainstream CVD epidemiology	Disability-related gap	Consequence for validity and equity	Proposed remedy (ref.)
Linked-data analytics	UK Biobank, All of Us, AIHW-linked admin data; >220,000-person cohorts; long-horizon outcome ascertainment	Disability not captured as a structured variable; inconsistent definitions across jurisdictions; disability-related missingness undocumented	Sparse data on disability sub-populations; biased prevalence and incidence estimates; under-powered sub-group analyses	Mandate ICF-aligned identifiers; FAIR governance (32)
Polygenic risk scores	Refined genetic stratification of coronary artery disease and atrial fibrillation; entering primary-prevention trial protocols	PRS calibration not reported for disability sub-groups; rare-variant architectures in syndromic CVD (trisomy 21, 22q11.2) under-studied	Mis-calibrated risk in syndromic CVD; under-treatment in genetic high-risk disability populations	Disability-stratified PRS validation; rare-variant analyses (2, 3)
Target trial emulation	Defensible causal inference from observational EHR data; standardised handling of immortal-time bias	Few emulations in disability populations; eligibility criteria often inherit trial-exclusion patterns	Causal effect estimates for preventive interventions cannot be generalised to PwD	Emulate trials in linked NDIS-Medicare-PBS data; pre-register disability-inclusive protocols (17)
AI in cardiovascular care	CNNs on ECG and CMR; transformers on EHR time-series; gradient-boosted trees for tabular risk prediction	Training cohorts unrepresentative; disability-stratified fairness metrics rarely reported; algorithmic-bias audits absent	Degraded discrimination/calibration in disability sub-groups; allocation bias against high-need users	Disability-stratified AUROC/calibration; intersectional MAIHDA; TRIPOD + AI reporting (33, 34)
Wearable digital phenotyping	Continuous-time arrhythmia, sleep and activity phenotypes; integration with clinical records	Devices validated principally in younger, ambulant, non-disabled users; motion-artefact and user interface (UI) accessibility seldom evaluated	Missed events, false alarms; inequitable access to remote-monitoring care pathways	Co-design with disabled users; impairment-domain validation (4)

### 4.4 A layered architecture for disability-inclusive cardiovascular epidemiology

**Figure 2:**
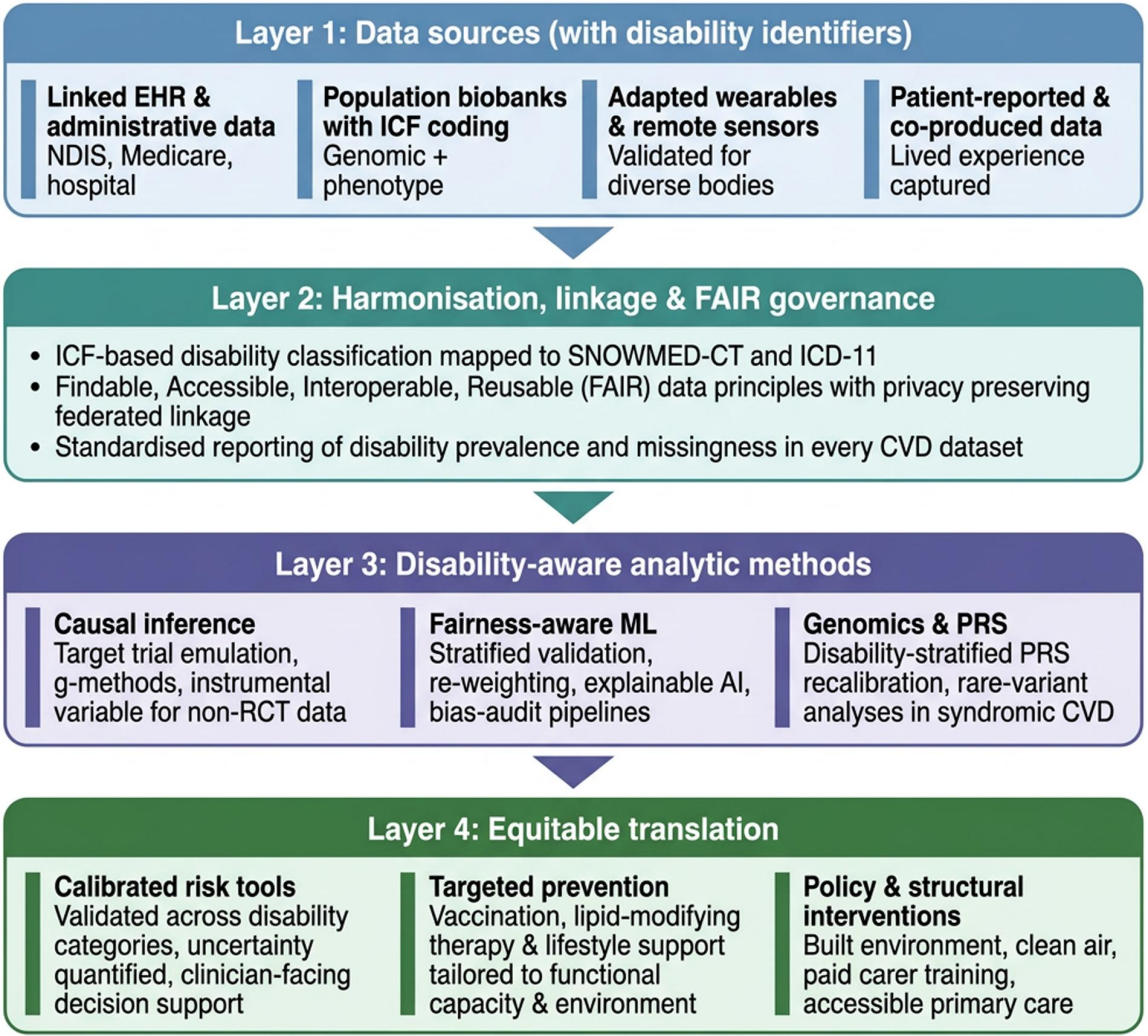
A four-layer architecture for disability-inclusive cardiovascular epidemiology, integrating inclusive data sources, FAIR-governed harmonisation, disability-aware analytic methods, and equitable translation.

This article proposes a four-layer framework (Figure 2) that adapts methods already validated in adjacent domains and ports them to the disability–CVD intersection.

**Layer 1 —Inclusive data sources:** International Classification of Functioning, Disability and Health (ICF)-aligned disability identifiers should be mandated in every CVD dataset, including clinical-trial enrolment registries, hospital admissions and biobank phenotypes. In the Australian setting this implies linkage of National Disability Insurance Scheme (NDIS) participant data with the Medicare Benefits Schedule, the Pharmaceutical Benefits Scheme and state-level hospital-admission data through the Multi-Agency Data Integration Project. Crucially, identifiers should be captured at the level of disability domain (physical, sensory, intellectual, psychosocial, neurodevelopmental, multiple) rather than as a single binary indicator, so that the heterogeneity described in Section 4.1 can be analytically respected. Wearable and remote-monitoring devices should be co-designed with disabled users and validated for signal quality across diverse body morphologies, mobility patterns and assistive technologies.

**Layer 2 — Harmonisation and FAIR governance:** The FAIR (Findable, Accessible, Interoperable, Reusable) data principles (32), should be adopted with privacy-preserving federated linkage. ICF disability classifications should be mapped to Systematized Nomenclature of Medicine—Clinical Terms (SNOMED-CT) and ICD-11 to enable interoperable, cross-jurisdictional analysis. Every cardiovascular dataset should report disability prevalence, the operational definition used and the proportion of disability-related missingness as standard metadata.

**Layer 3 — Disability-aware analytic methods:** Three method families are immediately portable. First, target trial emulation (17) enables causal inference from observational data on populations in whom randomised trials are infeasible, directly applicable to interventions in PwD. Second, fairness-aware machine learning, including stratified validation, instance re-weighting, *post-hoc* calibration and explainable AI, can detect and partially mitigate the algorithmic biases documented in Section 4.2 (30, 31). Specifically, fairness reporting should be expanded to include, for each disability sub-group: discrimination (area under the receiver-operating-characteristic curve, AUROC; area under the precision-recall curve, AUPRC); calibration (calibration-in-the-large, calibration slope, expected calibration error); confusion-matrix-derived rates (sensitivity, specificity, positive and negative predictive values, false-positive and false-negative rates); clinical utility (net benefit at clinically relevant decision thresholds via decision-curve analysis); and uncertainty quantification (prediction-interval coverage, conformal-prediction sets). Fairness must additionally be assessed across intersecting characteristics such as disability domain, sex, age, ethnicity, socioeconomic position and rurality using quantitative intersectional frameworks such as Multilevel Analysis of Individual Heterogeneity and Discriminatory Accuracy (MAIHDA) (34). Reporting of CVD prediction models that use regression or machine-learning methods should comply with the TRIPOD + AI statement (33). A concrete worked illustration is provided by Obermeyer and colleagues' audit of a widely deployed United States population-health risk-management algorithm, in which historical health-care expenditure had been used as a proxy for need; the algorithm systematically under-allocated additional care to Black patients, and recalibration with direct measures of need raised the proportion of identified high-risk patients from 17.7 per cent to 46.5 per cent (30). By direct analogy, using all-cause hospitalisation or non-cardiac claims expenditure as proxies for CVD risk in administrative data would systematically under-rank people with disability whose service use is dominated by disability-related rather than cardiovascular care.

Third, polygenic risk scores require disability-stratified recalibration; rare-variant analyses are particularly relevant in syndromic forms of cardiovascular disease, for example trisomy 21 and 22q11.2 deletion syndrome, in which risk architectures differ from those of the general population.

**Layer 4 — Equitable translation:** Risk-prediction tools must be calibrated and reported separately for people with disability, with quantified uncertainty. Preventive interventions, vaccination (23, 24), lipid-modifying therapy, blood-pressure control and physical-activity support, must be tailored to functional capacity and the environmental context, including access to accessible primary care and trained paid carers. Policy translation should target the structural environment, built-environment standards, clean-air policy (35), and the proactive inclusion of disability indicators in cardiovascular performance frameworks.

### 4.5 Implementation challenges and governance considerations

The framework above is deliberately ambitious; its implementation faces five categories of practical barrier that must be acknowledged and actively managed. First, definitional heterogeneity across jurisdictions: ICF coding, the Washington Group Short Set of Questions, the ICD-11 functioning supplement and national disability registers each define disability differently, with implications for prevalence estimates and cross-dataset comparability. Pragmatic crosswalks and shared metadata standards are required, with the Washington Group instrument offering a defensible interim standard for survey settings. Second, privacy and re-identification risk are amplified for small disability sub-populations, particularly in syndromic conditions with low prevalence; differential privacy, *k*-anonymity thresholds, and privacy-preserving federated learning approaches that keep raw data behind institutional firewalls and exchange only model parameters are credible mitigation strategies (36). Third, consent and supported decision-making require careful design, particularly for people with cognitive or communication disability. Article 12 of the United Nations Convention on the Rights of Persons with Disabilities establishes the right to equal recognition before the law, which in research practice translates into supported, not substituted, decision-making, accessible information materials and consent processes that respect functional capacity. Fourth, the risk of disability data being misused in insurance underwriting, employment screening, immigration adjudication or social-protection eligibility decisions is real and must be addressed through purpose-limited governance, data-use agreements, ethics-committee oversight and statutory anti-discrimination protection. Fifth, resource constraints in routine EHR capture mean that implementation cannot be solely a data-policy mandate; it requires investment in clinician training, EHR vendor specifications, primary-care workflow redesign and remuneration structures that reward accurate disability coding. The framework is therefore presented as a structured agenda for incremental adoption rather than a single transition.

## 5 Expanding the epidemiological lens: social, environmental and intersectional drivers

CVD epidemiology has rightly broadened its scope to encompass the social and environmental determinants of health. Air pollution is the most important environmental risk factor contributing to global CVD mortality and disability: Rajagopalan and colleagues report that short-term elevations in fine particulate matter (PM_2.5_) increase the relative risk of acute cardiovascular events by 1–3 per cent within days, and that longer-term exposures over several years increase risk by approximately 10 per cent (35). Climate change is now a recognised CVD risk multiplier: the 2024 *Lancet* Countdown reported record-breaking heat exposure, with ten of fifteen indicators of climate-related health hazards reaching new highs and substantial increases in heat-related mortality among adults aged 65 years and older (37). In Australia, this is manifest in documented increases in cardiovascular hospital admissions during heatwaves and an evolving evidence base on bushfire smoke as an acute cardiovascular trigger.

These determinants do not act uniformly. PwD are systematically over-exposed to ambient air pollution because of housing-market constraints, are under-served by public-transport networks that influence physical-activity opportunities and face elevated heat-related morbidity owing to medication interactions, thermoregulatory differences associated with some impairments and reduced capacity to relocate during extreme weather events. Intersectionality with race, ethnicity, gender, low income and rurality compounds these exposures. A genuinely transformative cardiovascular epidemiology must integrate environmental, social and disability variables within a single analytic frame rather than treating them sequentially.

### 5.1 Global perspective: disability-inclusive cardiovascular disease epidemiology in low- and middle-income countries

The evidence base summarised in this review is, however, disproportionately drawn from high-income settings, and the framework must be adaptable rather than imported when applied to low- and middle-income countries (LMICs), where approximately 80 per cent of the world's 1.3 billion people with disability live (7). The Banks, Kuper and Polack systematic review found a robust bidirectional association between disability and economic poverty across LMIC settings, with disability-poverty cycles amplifying cardiovascular risk through nutritional insecurity, household and indoor air pollution from solid-fuel cooking, occupational hazards and reduced access to primary care (38).

Four implementation challenges are particularly salient in resource-constrained settings. First, data infrastructure: linked EHR and population biobanks are scarce, health information systems are frequently fragmented and partly paper-based, and civil registration and vital statistics are often incomplete, which constrains both disability ascertainment and CVD outcome capture. Second, digital literacy and accessibility: digital-literacy gradients, the affordability of devices and connectivity, and accessibility barriers compound disability-related exclusion, although very high mobile-phone penetration in many LMICs offers a countervailing opportunity for mobile-first CVD surveillance. Third, healthcare accessibility: workforce shortages, out-of-pocket financing and geographic maldistribution of services limit access to diagnosis and secondary prevention, disproportionately for PwD. Fourth, AI implementation: models trained on high-income, predominantly European-ancestry populations may generalise poorly to demographically and epidemiologically different LMIC populations, risking biased and poorly calibrated predictions unless locally validated on representative data (39).

Concrete, transferable models already exist. Community-health-worker-delivered, smartphone-supported CVD risk screening has been deployed across several LMICs; the World Health Organization HEARTS technical package provides a standardised primary-care approach to CVD risk management suited to resource-constrained systems; and the Washington Group Short Set of Questions has been incorporated into national household surveys in more than 80 LMICs, offering a defensible operational standard for disability identification where formal ICF coding is unavailable. Mapped onto the four-layer framework, three adaptations are necessary: data-source layering should use whichever administrative or census instrument is available, with impairment-domain stratification approximating disability-stratified validation where ICF coding is absent; federated learning is particularly attractive where data-sovereignty or capacity considerations constrain cross-border data movement (36); and wearable and remote-monitoring deployments should favour low-cost, ruggedised, smartphone-tethered instruments over premium consumer wearables to achieve equitable coverage. Designed in this way, disability-inclusive CVD epidemiology becomes globally portable rather than a high-income preoccupation.

## 6 Conclusion: a research agenda for the next five years

The cardiovascular health of PwD is a discriminating test of contemporary epidemiology. The same big-data and AI tools currently celebrated for their capacity to refine precision medicine in the general population can, with deliberate methodological adaptation, close one of the field's most persistent and unjust evidence gaps. Five priorities follow from the preceding analysis:.

1. **Mandate disability identifiers:** Every CVD trial registry, biobank and major linked-data infrastructure should adopt ICF-aligned disability variables as standard metadata, with a defined transition period to full implementation.
2. **Disability-stratified validation as a publication standard:** Journals should require any cardiovascular risk-prediction or AI model to be validated and reported separately for people with disability, with calibration and discrimination metrics declared at submission, in accordance with the TRIPOD + AI reporting standard.
3. **Co-designed digital phenotyping:** Wearables, remote-monitoring platforms and decision-support tools should be co-produced with disabled users and validated across functional categories before deployment.
4. **Federated, privacy-preserving analytics:** National disability schemes, in the Australian setting, the NDIS, should be linked to cardiovascular outcome data through federated, privacy-preserving methods, enabling causal-inference and target-trial-emulation studies of interventions that are unethical or infeasible to randomise.
5. **Bias auditing of deployed AI:** Any AI tool used in cardiovascular care should undergo independent algorithmic-bias auditing across disability categories, with public reporting of findings and a defined remediation pathway when disparities are identified.

**Figure 3:**
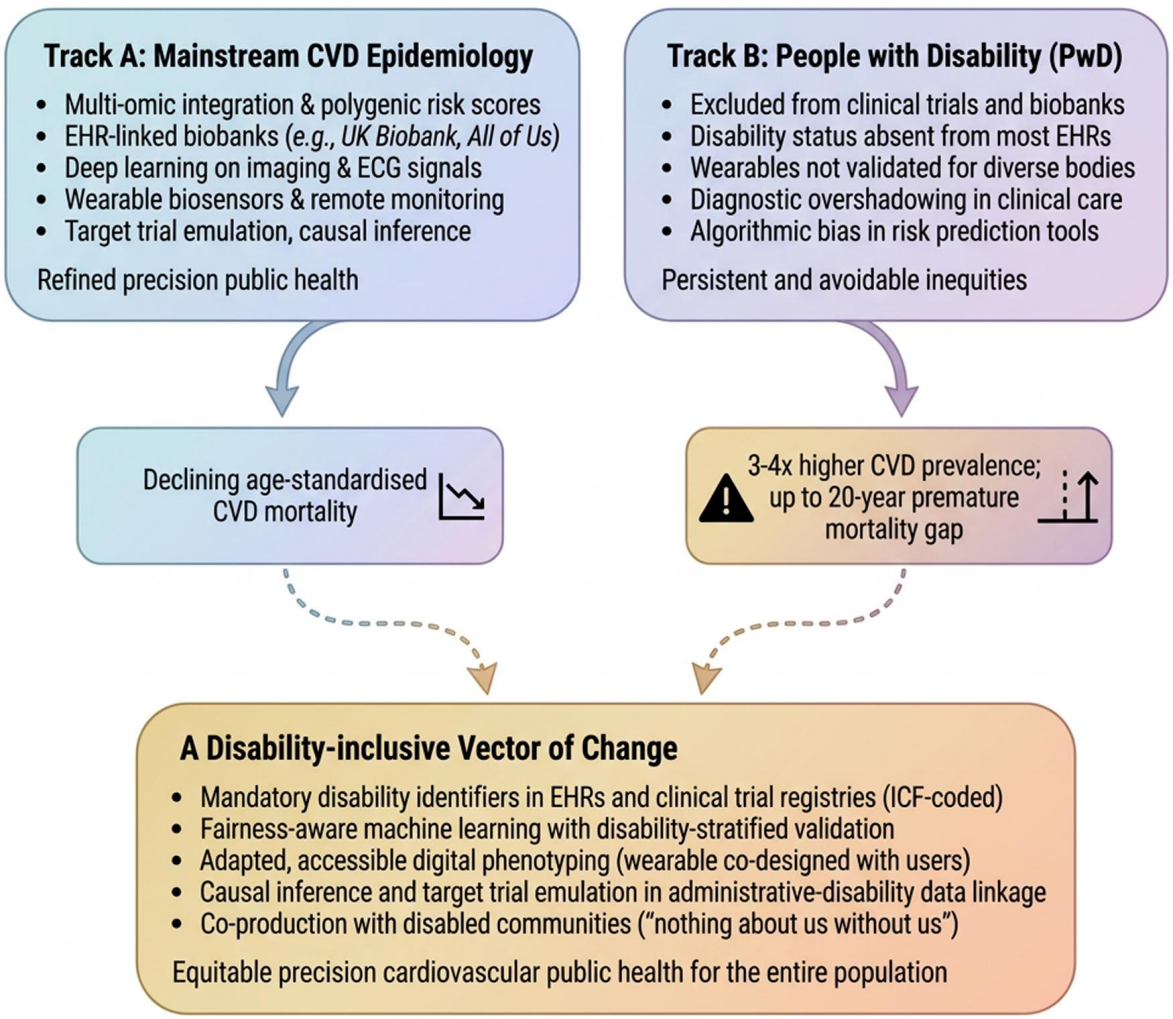
The two-track trajectory of contemporary cardiovascular disease epidemiology and the disability-inclusive vector of change required to converge them.

Figure 3 summarises the case made in this review: the field's current trajectory bifurcates into a mainstream track of accelerating progress and a disability track of persistent inequity, and an integrative, disability-inclusive vector of change is required to bring the two together. Transformation in epidemiology is not measured by what it can predict for the average patient; it is measured by whether it can equitably protect the cardiovascular health of every member of society. The methods are now available. The remaining barriers are structural, ethical and political, and the responsibility to address them sits with researchers, journals, funders and clinicians alike.

## Supplementary material

Supplementary Figure S1 PRISMA-style flow diagram of the evidence-identification process.
