# Supplementary figures and images for: Navigating a new era in cardiovascular disease epidemiology: big data, artificial intelligence and the imperative of disability inclusion
Source: Front Epidemiol. 2026 Jul 9;6:1871078. doi: 10.3389/fepid.2026.1871078 (PMC13391520; doi:10.3389/fepid.2026.1871078)

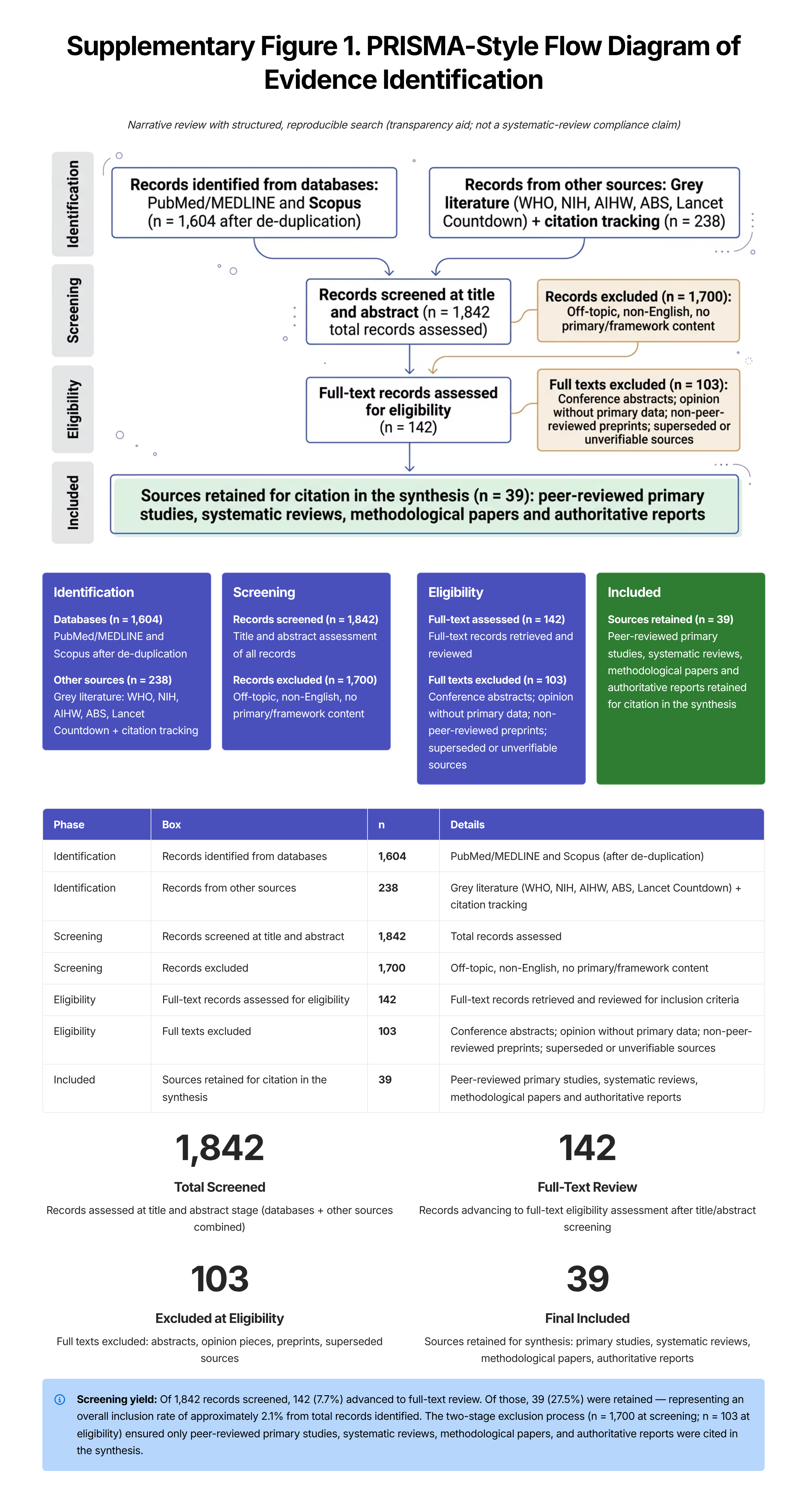

Supplement: Supplementary Figure S1 — PRISMA-style flow diagram of the evidence-identification process. [file Image1.tif]
